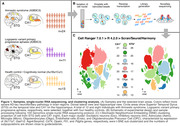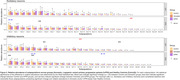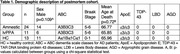# Single‐nuclei transcriptomic identifies type‐specific neuronal cell vulnerability in Amnestic and Logopenic Variant Primary Progressive Aphasia Alzheimer’s disease

**DOI:** 10.1002/alz.091703

**Published:** 2025-01-03

**Authors:** Felipe Luiz Pereira, Caroline Lew, Song Hua Li, Liara Rizzi, Igor Prufer Q C Araujo, Alexander V. Soloviev, Salvatore Spina, Jessica E Rexach, William W. Seeley, Claudia Kimie Suemoto, Renata Elaine Paraizo Leite, Kathy L Newell, Bernardino Ghetti, Melissa E. Murray, Lea T. Grinberg

**Affiliations:** ^1^ Memory and Aging Center, UCSF Weill Institute for Neurosciences, University of California San Francisco, San Francisco, CA USA; ^2^ Memory and Aging Center, Weill Institute for Neurosciences, University of California San Francisco, San Francisco, CA USA; ^3^ Memory and Aging Center, UCSF Weill Institute for Neurosciences, University of California, San Francisco, San Francisco, CA USA; ^4^ Department of Human Genetics, David Geffen School of Medicine, University of California Los Angeles, Los Angeles, CA USA; ^5^ Program in Neurogenetics, Department of Neurology, David Geffen School of Medicine, University of California Los Angeles, Los Angeles, CA USA; ^6^ Division of Geriatrics, Department of Internal Medicine, University of Sao Paulo Medical School, São Paulo, São Paulo Brazil; ^7^ Division of Geriatrics, University of São Paulo Medical School, São Paulo, São Paulo Brazil; ^8^ Physiopathology in Aging Laboratory (LIM‐22), Department of Internal Medicine, University of Sao Paulo Medical School, São Paulo, São Paulo Brazil; ^9^ Department of Pathology, University of Sao Paulo Medical School, São Paulo, São Paulo Brazil; ^10^ Indiana University, INDIANAPOLIS, IN USA; ^11^ Department of Pathology and Laboratory Medicine, Indiana University School of Medicine, Indianapolis, IN USA; ^12^ Mayo Clinic, Jacksonville, FL USA; ^13^ Physiopathology in Aging Laboratory (LIM‐22), University of São Paulo Medical School, São Paulo, São Paulo Brazil; ^14^ Memory & Aging Center, Department of Neurology, University of California in San Francisco, San Francisco, CA USA

## Abstract

**Background:**

Individuals meeting neuropathological criteria for Alzheimer’s disease (AD) may manifest with atypical clinical syndromes. Past work showed that the neurobiological basis for these differences is related to specific neuronal vulnerabilities for tau pathology. For instance, amnestic cases have a higher burden of neurofibrillary changes in CA1. In contrast, logopenic variant primary progressive aphasia (lvPPA) cases have a higher tau burden in the superior temporal gyrus (STG). Single‐cell technology enables investigations on the molecular basis of differential neuronal vulnerability in AD. Consequently, we delved into the factors that underlie this selective vulnerability by analyzing brain samples from individuals exclusively afflicted with AD but exhibiting diverse clinical manifestations.

**Method:**

snRNA Sequencing using the Chromium Single Cell 3′ (10X Genomics, USA) on nuclei cells extracted from the CA1 sector and posterior STG of postmortem brain tissue of 48 individuals either meeting pathological criteria for AD (A3B3C3; 24 amnestic and eleven lvPPA) and healthy controls (A≤1B≤1C≤1; n = 13) (Table 1, Fig. 1A/B). Bioinformatics analyses were conducted using Cell Ranger and R software. Comparisons between cell subpopulations were conducted with the Wald statistical test, and p‐values < .05 were considered significant.

**Result:**

After quality control, we recovered more than 250k nuclei with a mean of 2,130 genes per nuclei. Upon cross‐sample alignment and t‐stochastic neighborhood embedding clustering (Fig. 1C), we found 21 excitatory neuronal subpopulations (Exc‐sub) in CA1 and 26 in STG, and 22 and 25 inhibitory neuronal subpopulations (Inh‐sub) in CA1 and STG, respectively; 16 astrocytes subpopulations in both areas and 20 microglia subpopulations in CA1 and 17 in STG (Fig. 2). One STG Exc‐sub, expressing CUX2 and LAMP5 genes showed vulnerability in lvPPA patients. Also, one STG Inh‐sub, expressing the ADARB2 gene, showed vulnerability for all AD patients.

**Conclusion:**

Our preliminary study identified a vulnerable population of excitatory neurons related to lvPPA. We are conducting validation studies using quantitative pathology to confirm these results. Furthermore, analysis of a higher number of cases is ongoing and will continue to inform on factors associated with neuronal vulnerability.